# Child Sexual Abuse and Associated Factors Among High School Female Students in Arba Minch Zuria Woreda, Southern Ethiopia: A Cross-Sectional Study

**DOI:** 10.1007/s13224-024-01986-6

**Published:** 2024-04-06

**Authors:** Habtamu Wana Wada, Kassahun Fikadu Tesema, Gebremaryam Temesgen, Dagne Deresa Dinagde

**Affiliations:** 1 Department of Midwifery, Regional College of Health Sciences, Arba Minch, Ethiopia; 2 Department of Midwifery, College of Health Sciences, Arba Minch University, Arba Minch, Ethiopia; 3 Department of Midwifery, College of Health Sciences, Mattu University, Mettu, Ethiopia

**Keywords:** Child sexual abuse, Carnal abuse, High school, Arba Minch, Southern Ethiopia

## Abstract

**Background:**

Child sex abuse is a severe violation of fundamental human rights that has numerous negative consequences, including physical and psychological ones, and is a huge global public health issue. It is a significant public health issue that affects millions of people annually around the world but is the least acknowledged and reported form of violence in Ethiopia against schoolgirls. Thus, this study is very important to provide recent information on the magnitude of sexual abuse and its associated factors.

**Objective:**

This study was aimed at assessing the prevalence of lifetime child sexual abuse and associated factors among high school female students in Arba Minch Zuria Woreda (AZW), Southern Ethiopia.

**Methods:**

An institutional-based cross-sectional study was conducted among 414 randomly selected female high school students in Arba Minch Zuria Woreda between December 20, 2022, and January 20, 2023. A stratified and multi-stage sampling technique was used to select participants from selected high schools. A pretested, self-administered questionnaire was used, and the data were entered using EpiData version 4.6 and analyzed using SPSS (the Statistical Package for Social Science) version 27. Bivariate and multivariate analyses were carried out to identify factors associated with sexual abuse using binary logistic regression. Statistical significance was set at a *p*-value of less than 0.05.

**Result:**

In this study area, prevalence of child sexual abuse was 45% (95% CI 40.6–50.2). Students who slept alone at home (AOR = 4.93, 95% CI 1.46–16.67); having no open discussions with their parents (AOR = 4.49, 95% CI 1.62–12.47); drinking alcohol (AOR = 3.3, 95% CI: 1.32–8.28); and having friends who drink alcohol or chew chat (AOR = 6.14, 95% CI 1.58–23.9) were the identified factors associated with child sexual abuse.

**Conclusion:**

This study found a high level of child sexual abuse among high school female students in Arba Minch Zuria Woreda. High schools must collaborate with parents to develop a strategy to reduce sexual abuse acting against human rights, focusing on identified factors.

**Supplementary Information:**

The online version contains supplementary material available at 10.1007/s13224-024-01986-6.

## Introduction

### Background

Globally, the prevalence of CSA among girls is estimated to be 19.7% [1]. As compared to Europe and Africa (9.2 and 10.1%), the frequency of CSA in Asia and Delhi (23.9 and 27%) was higher [2, 3]. According to the international and regional legal framework, a child is defined as any individual who is less than the age of 18, unless the majority age is earlier under the applicable law [4]. Child sexual abuse involves using children for an adult's sexual enjoyment, is a crime against children, and is arguably one of the least recognized and least detected types of child abuse in the world [5].

Contact and non-contact child sexual abuse are the two main categories that are usually identified. Abuse of contacts includes actions involving penetration (such as rape and oral sex). Non-contact activities include forcing or threatening children to view or create pornographic material, watching sexual activity, encouraging children to act or behave inappropriately sexually, and grooming a child in order to abuse them and children who were disabled or in residential care were at high risk. Children with mental or intellectual disabilities are more at risk than those with other disabilities when it comes to disabilities [6]**.** People of all ages and genders experience sexual assault in developing countries, but children and adolescents are the most common victims, and these incidents frequently have major repercussions [7, 8]**.**

Around the world, one in twenty teenage girls experienced compulsion when they had their first sexual encounter. When sexual assault (rape) occurs on or near school property, there may occasionally be more overt aggression involved [1]. In Sub-Saharan Africa (SSA), child sexual abuse is a common problem. Victims have been known to be sexually abused by family members, neighbors, and other acquaintances. A local poll on child sexual abuse prevalence found that 34.4% of female respondents had been sexually abused as children [2]**.** According to research among female students in southern Nigeria, 26% [9], one of the highest incidences of sexual assault worldwide occurs in South Africa, where teenage girls between the ages of 12 and 17 are especially vulnerable (39%) [10]

In the context of Ethiopian high schools, studies were done on the magnitude of CSA in Butajira, which was 32.8% [11], in Gandhi Memorial Hospital in Addis Ababa, which was 42.7% [12], in Bahir Dar, which was 37.3% [13], and in Dire Dawa, which was 48.9% [7]. According to a WHO report, in many underdeveloped nations, including Ethiopia, CSA is the least recorded form of violence, with barely one out of ten instances being reported [14].

Child sexual abuse left the victim with feelings of fear, blame, fury, guilt, and/or disbelief, all common psychological responses among child sexual abuse victims. As a result, they suffer frequently from the post-traumatic syndrome, including the burden of STDs, HIV/AIDS, unsafe abortion, unwanted pregnancy, tears, and bleeding [15]. An estimated 74 million unwanted pregnancies occur worldwide each year, which results in 25 million unsafe abortions and 47,000 maternal deaths annually [16]. However, about 2.9 million American women (2.4%) become pregnant as a result of rape at some point in their lives [17]. A comprehensive solution is necessary to address the complex societal problem of CSA. Poor governance, culture, weak rule of law, unemployment, social and gender norms, gender inequality, low income, restricted educational opportunities, and the absence of one or both parents are all highly associated with sexual abuse [2, 9].

Additionally, few studies have shown long-term negative impacts on individuals. Mental health problems in childhood are shown to have a much greater impact on long-run economic outcomes when compared to physical health problems [18].

In particular, Goal 16.2 of the Sustainable Development Goals (SDGs) places a strong emphasis on the rights and well-being of children; the target states decreasing the risk of violence against children [19]. This is a rare chance to realize every child's right to live in a world free from violence and fear [20]. The Ethiopian government has established plans to provide comprehensive sexual and reproductive health in line with the SDG 2030 objective. In the Health Sector Transformation Plan II, information, counseling, and services are recognized as critical tactics to enhance adolescent and youth health [21]. This helps to reduce child physical injury and child sexual abuse by 70% [22]. Additionally, school-based sex education is essential for young people's sexual health and well-being. According to the findings of three decades of research, sex education in primary schools has the ability to significantly reduce child sexual abuse in addition to preventing pregnancy and STIs [23].

In 1991, Ethiopia ratified the UNCRC. Since then, the government has undertaken a wide range of initiatives to guarantee the protection and advancement of children's rights and well-being. According to the 2004 Ethiopian amended law (Article 623(2)(a)/2004), rape or other abuse of a girl between the ages of 13 and 18 by a member of the opposite sex constitutes an aggravating circumstance that is punishable by up to 20 years in jail.

Approximately 48% of Ethiopia's population is comprised of adolescents, who require special care during childhood. Thus, schoolgirls between the ages of 10 and 19 are considerably more vulnerable to sexual abuse, child trafficking, and other associated issues than adults in Ethiopia [24]. This means child sexual abuse continues to be a serious public health problem. Furthermore, it is common for underreporting; little study has been done on the magnitude of CSA among high school students in the study area, which is already old. Therefore, studying child sexual abuse is crucial to providing an evidence-based intervention, and thus, this study was conducted to assess the magnitude and associated factors of child sexual abuse (CSA) among female high school students in Arba Minch Zuria Woreda in 2023.

## Methods and Materials

### Study Design and Setting

An institution-based cross-sectional study was conducted in Arba Minch Zuria Woreda among high school female students attending regular education from December 20, 2022, to January 20, 2023. Arba Minch town is located 505 km southwest of Addis Ababa, the capital city of Ethiopia, and 275 km away from Hawassa, the commercial and administrative center of the southern region. According to the 2022 population projection, total populations of about 129,666 people were living in 2023. It is one of 19 Woreda that are found in the Gamo zone, which has seven high schools that provide services for regular and irregular classes. The total number of students in all high schools is 3132, and the total number of female students is 1639.

### Study Participants

High school female students who satisfied the inclusion and exclusion criteria were randomly selected from the source population to participate in the study. The study's source population comprised all female high school students enrolled in classes during the academic year 2022/2023.

### Eligibility Criteria

Due to the difficulty in gathering the necessary data, students who were critically ill, students who attended classes at night, and students who attended classes on the weekends were not included in the study. Those who matched the requirements but declined to provide their permission to take part in the study were also excluded.

### Sample Size Determination

The sample size was calculated using single population proportion formula based on the following assumption as population proportion (*p* = 8.7%) was taken from study conducted at Wolaita Sodo, Southern Ethiopia [25], with margin of error *d* = 5%.

$$ n = z^{2} \frac{{p\left( {1 - p} \right)}}{{d^{2 } }}. $$

Therefore *n* = (1.96)2*0.087(0.913), *n* = 122*3(DE)/(0.05)^2^ = 366, where *n* = sample size, DE = design effect.

*z* = standard distribution value for 95 CI, *z* = 1.96.

*p* = proportion of sexual abuse 8.7%, *d* = marginal error = 0.05(5%), DE = 3.

By adding 15% non-response rate = 54.9 + 366 = 421 were total sample size.

### Sampling Procedure and Technique

**Fig. 1:**
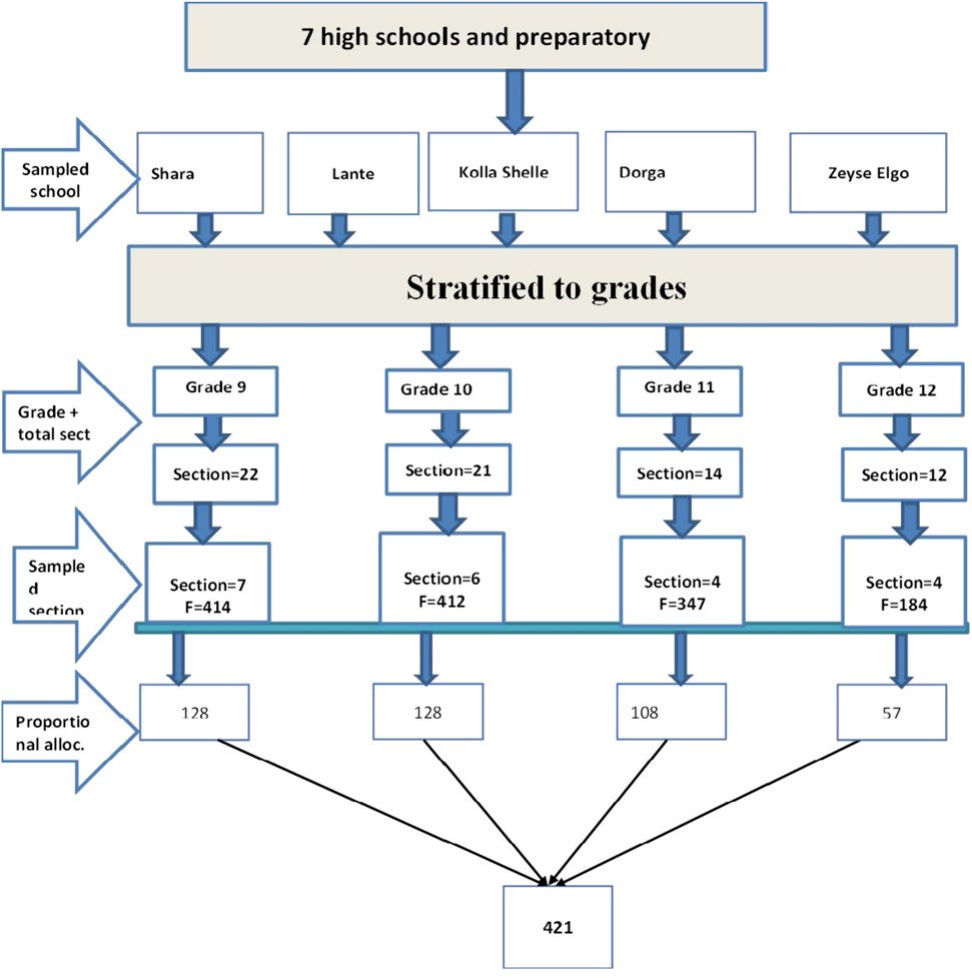
Schematic presentation of sampling procedure of child sexual abuse and associated factors at Arba Zuria Woreda (*n* = 421), 2023

Stratified and multistage sampling techniques were employed in this study by considering the variation seen at the students’ level of school and section. First, seven high schools around Arba Minch Zuria Woreda were identified, and a lottery method was applied. Five high schools were selected from which sections were taken. Then all sections in five selected schools were stratified according to their grade (Grade 9 as strata 1, Grade 10 as strata 2, Grade 11 as strata 3, and Grade 12 as strata 4). A list of female students in each grade (9th–12th) and sections was taken from all the selected schools. Among the five chosen schools, 21 sections were chosen from a total of 69 sections, using 30% as representative. The total sample size was then proportionally allocated among the five strata based on their total number of students. Participants from all of the selected schools and sections were finally included in the study using a simple random sampling procedure (Fig. 1).

### Data Collection Procedures

Five female teachers were selected to serve as data collectors and supervisors. Based on their prior data collection experience and the ability to communicate in local languages, data collectors and supervisors were chosen. Two days of ongoing training on the study's objectives, data collection instruments, and data collection techniques were provided to data collectors and supervisors. Data were collected by a self-administered questioner at break time in the morning and afternoon without affecting teaching and learning processes. To maintain privacy, a separate space was prepared in advance for students, with data collectors and facilitators assigned to each room. The questionnaire should be filled out and placed on the designated table, but participants were instructed not to write their names or any other identifying information on it. Then the investigator oversaw the procedure for gathering data. As soon as the data were obtained, the supervisors and investigators checked it for accuracy, clarity, and consistency.

## Study Variable

### Dependent Variable

- Child sexual abuse (yes/no).

### Independent Variables

- Socio-demographic factors: Age, grade, marital status, religion, residence, ethnicity, monthly of income.
- Family-level factors: Family size, parent educational occupation, Living arrangement, presence of parents.
- Individual factors: Having boyfriend, number of boyfriend, history of sexual intercourse, age at first sexual practice, number of sexual partner, SRH open discussion with parents, substance use and child’s health history drinking alcohol, chewing chat, and peers drink alcohol/chew chat.

### Variable Measurements

**Child sexual abuse:** There are six items measuring child sexual abuse and a student who reported at least one items is regarded to have CSA and those exposed to CSA were recoded to Yes’1’or No’0’ [7].

**The physical forms of sexual abuse:** include genital contact, fondling, rape, and the use of a.

child for pornography [12].

**Non-physical forms of sexual abuse:** include indecent exposure, plain talk about sex designed to shock a child or arouse her curiosity and allowing the child to watch or hear sexual acts or materials [26].

**Rape:** is a kind of sexual assault that typically involves sexual contact or other types of sexual penetration against a person without that person's consent [7].

**Substance abuse:** is the use of alcohol, chat, and/or cigarette in quantities or ways that are harmful to the user or others [27].

**Alcohol drinking:** This information relates to alcohol consumption before and after abuse occurred, both by the perpetrators and the victims.

**Chewing chat:** This information relates to whether the abuse victims or the offenders used to chew chat before and after the abuse happened.

### Statistical Analysis

Before the two weeks of real data collection, 5% of the female students at Chencha Woreda High School completed pretests of the questionnaires. In response, adjustments were made to the instrument, clarity, and ambiguity of the language tool. After being modified, coded, and input into the EpiData version 4.6 software, the data were ready for analysis. Descriptive statistics, which comprised relevant features including frequencies, proportions, and summary statistics, were used to characterize the study population. The existence of a relationship between the dependent and independent variables was evaluated using binary logistic regression. For the multivariate analysis, a variable from the bivariate analysis with a *p*-value of less than 0.25 was chosen. The Hosmer–Lemeshow test (> 0.05) and the VIF (< 10) tests were used to assess multicollinearity and the model's goodness of fit, respectively. The adjusted odds ratio (AOR) and 95% confidence interval (CI) were used to determine the components related to the outcome variable. The threshold for statistical significance was set at a p-value of less than 0.05.

### Ethics Statement

Ethical clearance was obtained from the Institutional Review Board of Arba Minch University, College of Medicine and Health Science, with reference number IRB/HW1332/2022, and permission was obtained from the school directors. Every study participant provided written informed consent, or in the case of minors, consent was obtained from the children's parents or legal guardians. Data confidentiality was maintained at every stage. The freedom of study participants to leave the study at any time was upheld. Privacy was guaranteed, and no personal information was recorded on any study-related material. Unless otherwise agreed, no one was obligated to take part. The study was conducted according to regulations and guidelines for research involving human beings.

## Results

### Socio-Demographic Characteristics of Study Participants

Out of the expected 421 participants, 414 female students correctly completed the questionnaire, resulting in a 98.3% response rate. Due to grossly incomplete and inconsistent responses, seven respondents were excluded. One hundred eighty-one (43.7%) of those participants were between the ages of 16 and 17. The mean age of the total respondents was 17.19 ± 1.45 standard deviation. Of the total of study participants, 156 (35.3%) were from grade nine. Three hundred seventy-one (89.6%) were single. One hundred nineteen (28.7%) and 86 (20.8%) of respondents' mothers had a grade 1–4 education and were unable to read or write. More than half of the total participants, 275 respondents (66.4%), receive financial or educational assistance from their parents (Table 1).

**Table 1:** Socio-demographic characteristics of respondents on the study of the magnitude of CSA and its associated factors among high school female students in Arba Minch Zuria Woreda, Southern Ethiopia, 2023 (*n* = 414)

Variables	Alternative responses	Frequency	Percent %
Age	≤ 15	57	13.8
	16–17	181	43.7
	18–19	152	36.7
	≥ 20	24	5.8
Marital status	Unmarried	371	89.6
	Married	43	10.4
Residence	Urban	77	18.6
	Rural	337	81.4
Religion	Orthodoxy	111	26.8
	Muslim	57	13.8
	Protestant	211	51.0
	Other	35	8.5
Ethnicity	Gamo	246	59.4
	Wolaita	58	14.0
	Amhara	73	17.6
	Other*	37	8.9
Father occupation	Govt employee	79	19.1
	Daily worker	86	20.8
	Merchant	89	21.5
	Farmer	160	38.6
Father educational level	Unable to read and write	68	16.4
	Grade 1–4	76	18.4
	Grade 5–8	104	25.1
	Grade 9–12	100	24.2
	12 and above	66	15.9
Mother educational	Unable to read and write	86	20.8
Status	Grade 1–4	119	28.7
	Grade 5–8	119	28.7
	Grade 9–12	65	15.7
	12 and above	25	6.0

*Gofa, Oromo, Gurage, Konso

### Family-Level Character of Respondent

In terms of living arrangements, the students reported that 197 (46.6%) of them were living with both parents. Only 106 (25.6%) of the participants slept alone at home. More than half of the total respondents, 235 respondents (56.8%), have more than five family sizes. Three hundred fifty-six (86%) of the total study participants reported that their family monthly income was greater than 1317 ETB (Table 2).

**Table 2:** Family-level character of respondents on the study of the magnitude of CSA and its associated factors among high school female students in Arba Minch Zuria Woreda, Southern Ethiopia, 2023 (*n* = 414)

Variables	Alternative response	Frequency	Percent %
Living with parents	Yes	321	77.5
	No	93	22.5
Living status	Shared both parents	197	46.6
	Single parent	95	22.9
	Sharing with friends	50	12.1
	Alone	72	17.4
Co-sleeping	Mother	97	23.4
	Sister/s	191	46.1
	Brother/s	20	4.8
	Alone	106	25.6
Family size	< 5	179	43.2
	> 5	235	56.8
Caregivers conflict	Yes	144	34.8
	No	270	65.2
Substance use	Yes	129	31.2
Caregivers	No	285	68.8
Family monthly income	< 1317 > 1317	58 356	14.0 86.0
Supporting person	Parents	275	66.4
	Relatives	45	10.9
	Siblings	60	14.5
	Husband/boyfriend	34	8.2

### Substance Use and Sexual and Reproductive History of Respondents

In this study, 284 (68.6%) participants had ever had a boyfriend, and 209 (50.5%) reported having a history of sexual intercourse. One hundred seventy-four (42.0%) of the study subjects had open discussions with their parents about sexual and reproductive health issues. Concerning substance utilization, 164 (39.6%) respondents drank alcohol (Table 3).

**Table 3:** Substance use and sexual and reproductive history on the study of the magnitude of CSA and its associated factors among high school female students in Arba Minch Zuria Woreda, Southern Ethiopia, 2023 (*n* = 414)

Variables	Alternative response	frequency	Percentage (%)
Have a boy friend	Yes	284	68.6
	No	130	31.4
Number of a boy friend	Only one	206	72.5
	Two and above	78	27.5
Sexual intercourse history	Yes	209	50.5
	No	205	49.5
Based on your will	Yes	120	57.4
	No	89	42.6
Age at sex started	14–16	98	46.9
	17–19	102	48.8
	≥ 20	9	4.3
Number of sexual partners	Only one	111	53.1
	Two and above	98	46.9
Open discussions on SRH	Yes	174	42.0
	No	240	58.0
Alcohol drunk	Yes	164	39.6
	No	250	60.4
Currently drunk	Yes	152	92.7
	No	12	7.3
Frequency	Once weekly	36	22.0
	Twice weekly	48	29.3
	Once monthly	58	35.4
	Twice monthly	22	13.4
Chat chewed	Yes	75	18.1
	No	339	81.9
Frequency	Once weekly	16	21.3
	Twice weekly	21	28.0
	Once monthly	31	41.3
	Twice weekly	7	9.3
Friends drink alcohol/chat chat/both	Yes No	206 208	49.8 50.2

### Magnitude of Child Sexual Abuse of Participants Experienced

**Fig. 2:**
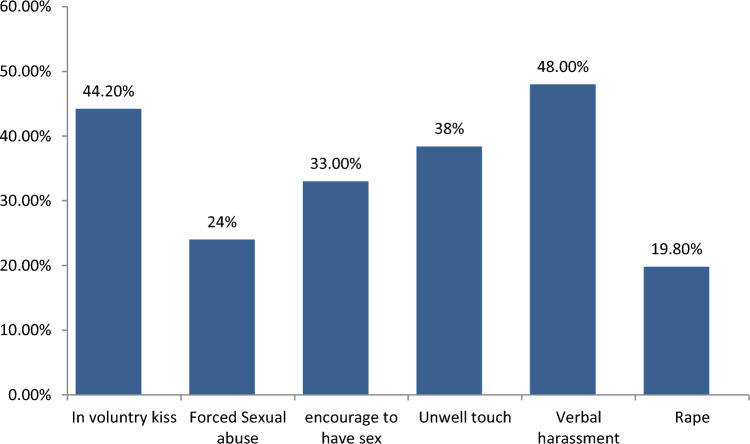
Types of child sexual abuse experienced among high school female students in Arba Minch Zuria Woreda in 2023 (*n* = 414)

Among the total respondents, the proportion of female students who reported at least one form of child sexual abuse was 186 (44.9%) (95% CI 40.6–50.2). Concerning the types of sexual abuse see (Fig. 2), respondents experienced the most: Forty-one (48%) of participants reported verbal harassment (a verbal form of sexual abuse), 38 (44.2%) reported involuntary (unwelcome) kissing, 33 (38.4%) of them reported unwelcome touch or body contact, 29 (24%) of students reported being forced to look at sexual activates, 28 (33%) of them respondents reported being encouraged to behave sexually, and 17 (19.8%) of the study participants reported rape.

### Reproductive Health Consequences Child Sexual Abuse (Rape)

Out of the total (17 survivors), 4 (23.5%) were pregnant unintentionally, and 23.5% had an abortion. STI (vaginal discharge) was also experienced by others (2, 11.8%), and 29.4% of women experienced vaginal bleeding right away following a rape. The majority of the respondents were raped by their boyfriend, 23.5%, only 6% by a family member, and 18% by an unknown person. The majority of the rape happened at the perpetrator’s’ home, 42%. About half of respondents reported only to their friends, and only two respondents reported to a legal body.

### Factors Associated With Child Sexual Abuse

After the assumptions of binary regression were checked, including visual inspection of the histogram-Q plot, outliers box, residual errors, multicollinearity, and Shapiro test, those variables in the bivariate analysis that were statistically associated with child sexual abuse with a *p*-value of less than 0.25 were exported to multivariate logistic regression to identify the association factors. Thus, having no parent–adolescent communication about sexuality and reproductive health, students, who drink alcohol, have a friend who drinks alcohol or chews chat, and living conditions (with whom they slept together in their home) were statistically significant (Table 4).

**Table 4:** Multivariate logistic regression output on factors associated with child sexual abuse among high school female students in Arba Minch Southern Ethiopia, 2023 (*n* = 414)

Variables	Responses	CSA		COR (95% CI)	(AOR 95% CI)	*P*-value
		Yes	No			
Living with	Both parents	72	125	1	1	0.56
	Single parents	33	62	0.92 (0.56–1.54)	0.56 (0.51–1.2)	–
	Friends	28	22	2.21 (1.18–4.15)	0.9 (0.82–2.4)	–
	Alone	53	19	4.84 (2.66–8.82)	1.2 (1.04–1.82)	–
Co-sleeping	Mother	34	63	1	1	
	Sister/s	78	113	0.1.28 (.77–2.12)	2.34 (0.73–7.49)	
	Brother/s	3	17	0.327 (0.09–1.19)	0.56 (.54–1.22)	
	Alone	71	35	3.76 (2.1–6.72)	4.93 (1.46–16.67)*	0.000*
Open discussion	Yes	26	148	1	1	0.006*
	No	160	80	11.4 (6.94,18.7)	4.49 (1.62–12.4)	
drunk alcohol	Yes	112	52	5.12 (3.34–7.85)	3.3 (1.317–8.28)*	0.000*
	No	74	176	1		
friends drink	Yes	139	67	11.4 (6.61–19.7)	6.14 (1.56- 23.9)*	0.000*
Alcohol (chewing)or both	No	47	161	1		
Chewed chat	Yes No	50 136	25 203	3.0 (1.76–5.12)	1.31 (0.9–1.9)	0.125

Students who did not have open discussions with their parents on sexual and reproductive health were about 4.5 times more likely to experience sexual abuse as compared to those students who had an open discussion with their parents on sexual and reproductive health issues (AOR = 4.49, 95% CI = 1.62–2.47). Those students who drink alcohol were 3.3 times (AOR = 3.3, 95% CI = 1.32–8.28) more likely to expose sexual abuse when compared with those who did not drink, and students who were having friends drink alcohol or chew chat were 6 times (AOR = 6.14, 95% CI = 1.58–23.9) more likely to experience lifetime sexual abuse than those who were not friends who drank alcohol or chew chat. Furthermore, the odds of experiencing lifetime sexual abuse among students who slept alone at home were five times higher than those who slept with their mothers (AOR = 4.93, 95% CI = 1.46–16.67) and more likely to experience lifetime child sexual abuse than those living with their mothers.

## Discussion

The findings from this study revealed that the lifetime magnitude of at least one form of sexual abuse was 44.9% (95% CI 40.6, 50.2). This finding is in line with studies conducted in different parts of developing countries, for instance, India, 47.0% [28], South East Nigeria, 42.1% [29], Dire Dawa town, 48.9% [7], and Bahir Dar city, 49.1% [13]

However, it is much higher than the study done in Butajira, 32.8% [11], Wolaita Sodo, 8.7% [25], Harar Regional State, 25% [30], Jimma, 31.9% [31], South Africa and Nigeria, 22.2% [9, 10], Addis Ababa,75.9% [26], West Shewa, 74% [32], and southern Brazil, 56% [33]. This variation might result from the diverse living circumstances, cultural norms, and values that different ethnic groups place on women and the difference may be due to the time of the study, sample size, and socio-demographic differences of the study participants.

Students who have not had open discussions with parents were at four times higher risk of experiencing CSA as compared to those who have had open discussions. This finding is consistent with a study done in Dire Dawa town, Arba Minch town, and Bahir-Dar town, where the odds of experiencing lifetime rape were much higher among students who never had open discussions than those who have had it [7, 13, 34]. This might be because open discussions with parents or guardians will disclose it and provide a chance to discuss its impacts on adolescent health. In this study, sexual abuse was significantly associated with students who drink alcohol being three times more exposed to child sexual abuse than those who do not. This finding was in line with the study conducted in Wolaita Sodo and Bahir Dar city [13, 35].

Students who had peers (male or female) who were drunken and chat chewing were six times more likely to experience CSA than those who were not friends' drunken alcohol or chewing chat. This finding was in line with the study conducted in Wolaita Sodo, Bahir Dar city, Harar regional state, and Dire Dawa town [7, 13, 30]. This could be explained by various mechanisms; at a behavioral or psychological level, alcohol may decrease the risk of perception and the ability to communicate assertively.

A study done in different settings revealed that lifetime experience of sexual abuse was associated with the living conditions of students, like with whom they live and with whom they sleep at home. Similarly, the findings of this study indicated that students who slept alone at home were four times more likely to experience child sexual abuse than those who slept with their mothers (parents). This finding is consistent with a study done among high school students in Dire Dawa town, Harar regional state, Arba Minch town, and South East Nigeria [7, 29, 30, 34]. One plausible explanation is that youngsters living with their parents are subject to close supervision and monitoring, which reduces the likelihood of them coming into contact with opportunistic predators.

### Limitations of Study

It is possible that there have not been enough reports of sexual assault incidents since our study topic deals with private and delicate sexuality-related issues. Due to these restrictions, the results of this investigation should be understood.

## Conclusion

This study found a high level of child sexual abuse in this study area. Factors significantly associated with child sexual abuse in this study were: having no open discussions about sexuality and reproductive health issues with parents; students drinking alcohol; having friends or peers who consume alcohol or chew chat; and the living conditions or arrangements of the participants. Hence, high schools must work with parents to establish a plan to decrease sexual abuse that violates human rights while emphasizing the noted factors.

## Supplementary Information

Below is the link to the electronic supplementary material.

Supplementary file1 (DOCX 49 KB)

## Data Availability

The dataset used in the current study are not available to the general public. Since not all participants gave us permission to publish their data, the raw data are still available from corresponding author (Mr. Habtamu Wana) with justifiable request.

## Abbreviations

AOR: Adjusted odds ratio
CI: Confidence interval
CSA: Child sexual abuse
SDGs: Sustainable development goals
SPSS: Statistical Package for Social Science
SSA: Sub-Saharan Africa
STIs: Sexually transmitting infections
