# Supplementary material for: Child Sexual Abuse and Associated Factors Among High School Female Students in Arba Minch Zuria Woreda, Southern Ethiopia: A Cross-Sectional Study
Source: J Obstet Gynaecol India. 2024 Apr 6;75(Suppl 1):52–61. doi: 10.1007/s13224-024-01986-6 (PMC12085442; doi:10.1007/s13224-024-01986-6)
Supplement: Supplementary file 1 — Supplementary file1 (DOCX 49 KB) [file 13224_2024_1986_MOESM1_ESM.docx]

**ANNEX 3; ENGLISH VERSION QUESTIONNAIRES**

Questioners were used in assessing the magnitude of child sexual abuse and associated factor. For each question, make a circle around a number that corresponds, and for the answers fill the blank space.

**Part 1. Socio-demographic characteristics of the child and their parents**

| S.No |  |  |  |  |
| --- | --- | --- | --- | --- |
|  | Variable | Responses | | |
|  |  |  | | |
| 1 | Age | **_________** | | |
|  |  |  | | |
| 2 | Grade | **_________** | | |
|  |  |  | |  |
| 4 | Marital status of respondents | 1.Single | |  |
|  |  | 2.Married | | |
|  |  |  |  | |
| 5 | Residence | 1. | Urban | |
|  |  | 2. | Rural | |
|  |  |  |  | |
| 6 | Religion | 1. | Orthodox | |
|  |  | 2. Muslim | | |
|  |  | 3.Protestants | | |
|  |  | 4.Others | | |
|  |  |  |  | |
| 7 | Ethnicity | 1. | Gamo | |
|  |  | 2. | Wolyta | |
|  |  | 3. | Amhara | |
|  |  | 4. | Others | |
|  |  |  |  |  |
| 8 | What is your father |  | 1. | Employee |
|  |  |  | 2. | Merchant |
|  |  |  | 3. | Daily laborer |
|  |  |  | 4. | Farmer |
|  |  |  |  |  |
| 9 | What is your mother |  | 1. | Unable to read and write |
|  |  |  | 2. | Grade 1–4 |
|  |  |  | 3. | Grade 5–8 |
|  |  |  | 4. | Grade 9–12 |
|  |  |  | 5. | Above grade 12 |
|  |  |  |  |  |

| 10 | What is your father |  | 1. | Unable to read and write |
| --- | --- | --- | --- | --- |
|  |  |  | 2. | Grade 1–4 |
|  |  |  | 3. | Grade 5–8 |
|  |  |  | 4. | Grade 9–12 |
|  |  |  | 5. | Above grade 12 |
|  |  |  |  | |
| 11 | Who supports you for learning / Source of | 1. | Parents | |
|  | Income | 2. Relatives | | |
|  |  | 3. | Self-supporting | |
|  |  | 4. | Husband/boyfriend | |
|  |  |  |  |  |

**Part.2 Family Level Factor**

| S.No | **Variables** | **Responses** | | |
| --- | --- | --- | --- | --- |
|  |  |  | | |
| 1 |  | 1. yes | | |
|  | Absence of one or both biological parent | 2.No | |  |
|  |  |  |  | |
| 2 |  | 1. | Both Parents | |
|  | Living arrangement of the child | 2. | Single Parents | |
|  |  | 3. | Freinds | |
|  |  | 4. | Alone | |
|  |  |  |  | |
| 3 | With whom do you sleep together in your | 1. | Mothers | |
|  | home**?** | 2. | Sisters | |
|  |  | 3. | Brothers | |
|  |  | 4. | Alon | |
|  |  |  | | |
| 4 | Family size | **________** | | |
|  |  |  | |  |
| 5 | The marital conflict between the child | 1.yes | |  |
|  | parents | 2.No | | |
|  |  |  | |  |
| 6 | Conflicts between parents | 1. | | Yes |
|  |  | 2. | | No |
|  |  |  | |  |
| 7 | Substance abuse by parents | 1. | | Yes |
|  |  | 2. | | No |
|  |  |  |  |  |

|  | Family monthly income | **___________** |
| --- | --- | --- |
|  |  |  |

**Part 3. Substance use and child’s health history**

| **So No** | **Variables** | **Responses** | | | **Remarks** |
| --- | --- | --- | --- | --- | --- |
|  |  |  |  |  |  |
| 1 | Have you ever had a boyfriend? | 1. | Yes |  | If No skip Q#2 |
|  |  | 2. | No |  |  |
|  |  |  |  | |  |
| 2 | How many boyfriends did you have in | 1. | Only one | |  |
|  | your life**?** | 2. | Two or more | |  |
|  |  |  |  |  |  |
| 3 | Have you ever had a history of sexual | 1. | Yes |  | If No skip |
|  | intercourse? | 2.No | |  | Q#4,5,6 |
|  |  |  |  |  |  |
| 4 | Was sexual intercourse based on your | 1. | Yes |  |  |
|  | will? | 2.No | |  |  |
|  |  |  | |  |  |
| 5 | Age at 1st sex | **______** | |  |  |
|  |  |  | |  |  |
| 6. | Lifetimes sexual partner | 1. one | |  |  |
|  |  | 2. two or more | | |  |
|  |  |  |  |  |  |
| 6 | Have you had any discussion with your | 1. | Yes |  |  |
|  | parents about SRH? | 2.No | |  |  |
|  |  |  | |  |  |
| 7 | Have you ever drunk alcohol? | 1 . Yes | |  | If no skip |
|  |  | 2. | No |  | question no 9,10 |
|  |  |  | |  |  |
| 8 | Are you drinking currently | 1 . Yes | | 2. No |  |
|  |  |  |  | |  |
| 9 | Alcohol drinking frequency | 1. | Once in a week | |  |
|  |  | 2. | Twice a week | |  |
|  |  | 3. | Once in a month | |  |
|  |  | 4. | Twice a month | |  |
|  |  |  | |  |  |
| 10 | Have you ever chewed Chat? | 1 . Yes | | 2. No | If no skip |
|  |  |  |  |  | question no |
|  |  |  |  |  | 11,12 |
|  |  |  | |  |  |
| 11 | Are you chewing currently? | 1 . Yes | | 2. No |  |
|  |  |  | | |  |
| 12 | Chat chewing frequency | 1.Once in a week | | |  |
|  |  | 2.Twice a week | | |  |
|  |  |  |  |  |  |

４０

|  |  |  |  | 3.Once in a month | |  |
| --- | --- | --- | --- | --- | --- | --- |
|  |  |  |  | 4.Twice in a month | |  |
|  |  |  |  |  |  |  |
| 13 | Do your friends drink alcohol (chewing) | | | 1 . Yes2. No | |  |
|  | or both? | |  |  |  |  |
|  |  |  |  |  |  |  |
| **4. Magnitude** and **Types of sexual abuse experiences** | | | | | | |
|  |  |  |  | |  | |
| So No |  | Variables | Responses | |  | |
|  |  |  |  | |  | |
| 1 |  | Have you had any | 1.Yes | |  | |
|  |  | child hood sexual | 2.No | |  | |
|  |  | Abuse you experienced? |  |  |  |  |
|  |  |  |  | |  | |
|  |  | If your answer is yes, | 1. Involuntary kissing | |  |  |
|  |  | select which types of child | 2. Forced to look at sexual | |  | |
|  |  | sexual abuse you were | activities | |  | |
|  |  | experienced | 3.Encourged to behave sexually | |  | |
|  |  |  | 4.Unwellcome touch | |  | |
|  |  |  | 5.Verbal harassment | |  | |
|  |  |  | 6.Rape | |  | |
|  |  |  |  | |  | |
| 2 |  | If your answer is rape | 1. Family member | |  |  |
|  |  | from above choice.by | 2.Schoomates | |  | |
|  |  | whom you raped | 3.Teachers | |  | |
|  |  |  | 4.Boyfriends | |  | |
|  |  |  | 5.Neigbors | |  | |
|  |  |  | 6.Strangers/unrecognized person | |  | |
|  |  |  |  | |  | |
| 3 |  | Place where it take | 1.survivors home | |  |  |
|  |  | place | 2.preparators home | |  | |
|  |  |  | 3.Hotel | |  | |
|  |  |  | 4.Inside the car | |  | |
|  |  |  | 5.public street/Field | |  | |
|  |  |  |  | |  | |
| 4 |  | Do you report it to | 1.Yes | |  |  |
|  |  | someone else? | 2.No | |  | |
|  |  |  |  |  |  |  |

４１

| 5 | If yes to whom you report | 1.Friends |
| --- | --- | --- |
|  |  | 2.Parets/Family |
|  |  | 3.Legal bodies |
|  |  |  |
| 6 | If no why did not report | 1.Fear of preprators |
|  |  | 2.Fear of family |
|  |  | 3.Fear of stigma |
|  |  | 4.I did not know what to do |
|  |  |  |
| 7 | Reproductive healthy | 1.Unwanted pregnancy |
|  | consequence of sexual | 2.Abortion |
|  | abuse | 3.Sexual transmitted disease |
|  |  | 4.Vaginally bleeding |
|  |  | 5.No |
|  |  |  |
